# The Role of IL-17 During Infections in the Female Reproductive Tract

**DOI:** 10.3389/fimmu.2022.861444

**Published:** 2022-04-14

**Authors:** Puja Bagri, Varun C. Anipindi, Charu Kaushic

**Affiliations:** ^1^ McMaster Immunology Research Centre, McMaster University, Hamilton, ON, Canada; ^2^ Department of Medicine, McMaster University, Hamilton, ON, Canada

**Keywords:** IL-17, FRT, infection, fungal, bacterial, HIV, HSV, mucosal

## Abstract

Interleukin-17 (IL-17A) is a cytokine involved in a complex array of both protective and detrimental processes. Although early biological studies focused on the pro-inflammatory function of IL-17 in the context of autoimmune and inflammatory disorders, it has become increasingly evident that the roles of IL-17 are far more nuanced. Recent work has demonstrated that the functions of IL-17 are highly context- and tissue-dependent, and there is a fine balance between the pathogenic and protective functions of IL-17. This is especially evident in mucosal tissues such as the female reproductive tract, where IL-17 has been shown to play an important role in the immune response generated during fungal, bacterial and viral infections associated with protection, but also with inflammation. In this review, we discuss the evolving landscape of IL-17 biology within the context of the vaginal mucosa, focusing on key findings that highlight the importance of this cytokine in genital mucosal immunity.

## Introduction

Interleukin 17A (IL-17A; referred to as ‘IL-17’ here) was cloned in the early 1990s and initially known as cytotoxic T lymphocyte-associated antigen 8 (CTLA-8) (1). Although first recognized as a new cytokine in 1995, the importance of IL-17 remained obscure until almost a decade later, when it was discovered that a novel population of CD4+ T helper (Th) cells, subsequently named Th17 cells, were characterized by their secretion of IL-17 (2–4). Following this discovery, the role of IL-17 has been examined in the context of many disease models.

**Figure 1:**
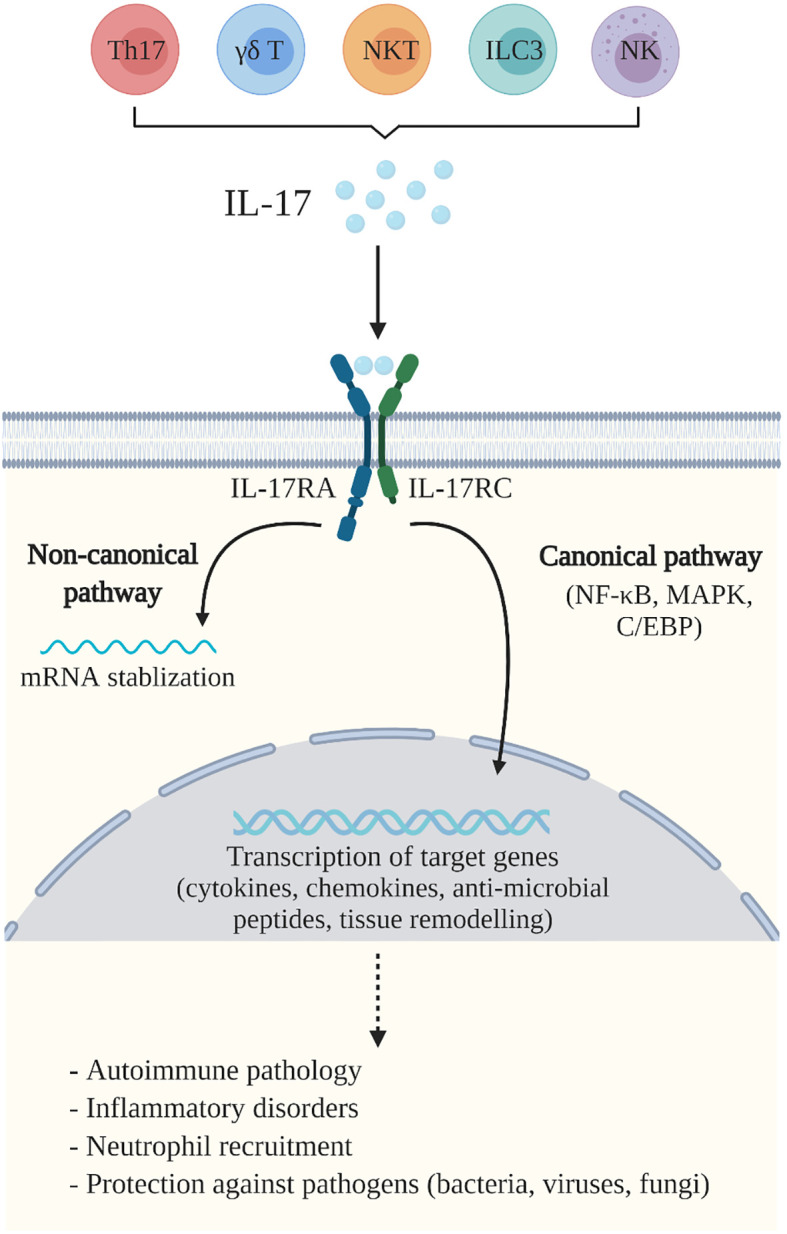
IL-17A-mediated immunity. IL-17A (IL-17) is produced by a variety of cells including Th17 cells, γδ T cells, natural killer (NK) cells, natural killer T (NKT) cells and group 3 innate lymphoid cells (ILC3). IL-17 binds to the IL-17 receptor (IL-17R)A and IL-17RC complex, expressed by a variety of cells including macrophages, fibroblasts, keratinocytes, and epithelial and endothelial cells. This initiates two primary IL-17 signalling pathways which mediate the essential functions of IL-17. The canonical pathway activates the nuclear factor kappa B (NF-κB), mitogen-activated protein kinase (MAPK) and CCAAT-enhancer-binding protein (C/EBP) pathways, that trigger transcriptional activation of downstream target genes, including pro-inflammatory cytokines, chemokines and anti-microbial peptides, as well as genes related to tissue remodeling. The non-canonical pathway leads to the stabilization of mRNA transcripts. Together, these pathways mediate immune responses which contribute to the pathogenesis of autoimmune and inflammatory diseases, neutrophil recruitment and are important for host defence against pathogens including bacteria, viruses and fungi. Created with BioRender.

The IL-17 family of cytokines includes six similarly structured ligands (IL-17A to IL-17F), of which, IL-17A and IL-17F are most closely related functionally. IL-17 cytokines signal through a dimeric receptor composed of pairs of five subunits: IL-17RA through IL-17RE (5, 6). IL-17 and IL-17F exist either as homodimers or as a heterodimer, and signal through an obligate dimeric IL-17RA and IL-17RC receptor complex (7). There are two primary signalling pathways initiated upon IL-17 binding to its receptor complex [reviewed in (5, 8)] (**Figure 1**). The first is a canonical pathway, which leads to the activation of nuclear factor kappa B (NF-κB), mitogen-activated protein kinase (MAPK) and CCAAT-enhancer-binding protein (C/EBP) pathways, and results in transcriptional activation of downstream, pro-inflammatory target genes. The second, noncanonical pathway, leads to the stabilization of mRNA transcripts which encode for intrinsically unstable targets including cytokines and chemokines. Overall, at the transcriptional and post-transcriptional level, IL-17 enhances the production of several immune mediators including chemokines, cytokines, antimicrobial peptides (AMPs) and other primarily inflammatory effectors (9).

IL-17 has several immunoregulatory functions, as detailed in a recent review (10). During infection, IL-17 is actively involved in neutrophil and monocyte recruitment through enhanced induction of various chemoattractants including CXCL1, CXCL2 and CXCL5 (11, 12). IL-17 is also known to induce granulocyte-colony-stimulating factor (G-CSF), which is involved in promoting the expansion and survival of neutrophils (13). Additionally, IL-17 plays an important role in maintaining epithelial barrier integrity by regulating the induction of AMPs during periods of homeostasis when the barrier is intact, and then by inducing immune mediators upon the loss of barrier integrity (14). Taken together, IL-17 is considered a key cytokine involved in the clearance of extracellular bacteria and fungi. However, in some cases aberrant IL-17 production can augment inflammation and cause tissue damage, which has been shown to occur in autoimmune or chronic inflammatory diseases such as rheumatoid arthritis (6).

The production of IL-17 is linked to several cellular sources. Traditionally, IL-17 is considered a cytokine secreted by Th17 cells, a subset of activated CD4+ T cells that secrete signature cytokines IL-17A and IL-17F, as well as IL-21 and IL-22 (12). Exposure of naïve CD4+ T cells to combinations of antigen-presenting cell (APC)-derived polarizing cytokines, including transforming growth factor-beta (TGF-β), IL-6, IL-21, IL-23 and IL-1β, leads to the differentiation of Th17 cells (15–18). Furthermore, Th17 cells are regulated by a master transcription factor, retinoic acid receptor-related orphan receptor gamma t (ROR-γt, encoded by Rorc), which induces the production of their signature cytokines (19). ROR-γt is also a major transcription factor associated with all subsets of IL-17-secreting cells, along with the transcription factor aryl hydrocarbon receptor (Ahr) (10).

In addition to Th17 cells, other cell types are also capable of producing IL-17. This includes innate and innate-like lymphocytes such as gamma-delta (γδ) T cells (20), natural killer (NK) cells, natural killer T (NKT) cells (21), lymphoid tissue inducer-like cells (LTi) and several populations of group 3 innate lymphoid (ILC3) cells (22, 23). These additional sources of IL-17 tend to accumulate at mucosal surfaces and have been shown to play an important role in the early immune response against pathogens, emphasizing the importance of IL-17 in innate immunity. Different pro-inflammatory cytokines including IL-1β and IL-23 are known to induce IL-17 production by these cells. Interestingly, γδ T cells in particular have been shown to be the primary source of IL-17 production in various settings of tissue homeostasis and infection [reviewed in (24)]. γδ T cells develop early in the fetus and provide immunity prior to the generation of adaptive immune responses (25). Unlike traditional T lymphocytes, γδ T cells can acquire their effector function during thymic development (25); thus, allowing them to produce baseline levels of IL-17 and/or respond early and more rapidly to pathogens compared to Th17 cells.

IL-17-mediated immunity has been demonstrated to play an important role in the immune response generated against pathogens in mucosal tissues including the female reproductive tract (FRT). The FRT is a unique mucosal site that is regulated by several factors in the microenvironment, including sex hormones and the vaginal microbiota [reviewed in (26–28)]. Importantly, the FRT is a critical site for enabling human reproductive success as well as protecting against sexually transmitted pathogens; thus, immune responses in this tissue must be well understood in order to help facilitate positive reproductive health. As such, we and others have focused on the role of this important immunoregulatory factor in the context of genital tract infections, and in this review, we will summarize and highlight the recent progress in the field of IL-17-mediated immunity during fungal, bacterial and viral infections in the FRT.

## Fungal Infections

Candida species are part of the normal microflora of the FRT but can become pathogenic under certain circumstances (29). Vulvovaginal candidiasis (VVC) caused by *Candida albicans* (*C. albicans*), is an opportunistic fungal infection that affects approximately 75% of healthy women of reproductive age globally at least once during their lifetime (30). Furthermore, following primary infection with *C. albicans*, 5-10% of women will subsequently experience recurrent infection (31), which is defined as experiencing at least 3-4 episodes yearly (32). Thus, VVC infection is a very frequent and distressing condition that can significantly impact quality of life.

Both human and animal studies have examined the role of IL-17-mediated immunity during *C. albicans* infection. Animal models are commonly used to study VVC pathogenesis (33), and the mouse model closely recapitulates the human disease. Although mice are not naturally susceptible to vaginal infection with *C. albicans*, treatment with estradiol (E2) enables persistent infection to occur. In several studies, IL-17 was seen to play a clear protective role in several types of mucosal candidiasis (i.e. chronic mucocutaneous and oropharyngeal candidiasis) and help regulate antifungal immunity by upregulating pro-inflammatory cytokines, neutrophil-recruiting chemokines and AMPS (11, 34–36). The role of IL-17 during VVC, however, remains unclear. For example, patients who have genetic defects in IL-17 do not show increased susceptibility to VVC as they do to other mucocutaneous forms of candidiasis (37, 38). Similarly, animal studies have shown that in the absence of IL-17 and/or IL-17-mediated signalling, mice demonstrated worsened disease outcomes following both systemic (39) and oral infection with *C. albicans* (34), and this was related to impaired neutrophil recruitment and AMP production. However, unlike candidiasis infections at other mucosal sites, neutrophils have been shown to be more damaging than protective during VVC (29, 40) or they appear to have a limited impact on fungal infection in the FRT. For instance, Yano et al. (41) reported that in the absence of IL-17 and other Th17-related cytokines, mice had similar levels of fungal burden following vaginal *C. albicans* infection compared to wildtype (WT) mice, suggesting IL-17 was not involved in response to infection. In addition, recent findings from Peters et al. (42) demonstrated that mice lacking IL-17RA did not exhibit altered VVC susceptibility, regardless of E2 administration, further supporting the idea that the Th17/IL-17 axis plays no role in the immunopathogenesis of VVC. In contrast, Pietrella et al. (43) found that when Th17 differentiation was inhibited during VVC challenge, there was greater exacerbation of disease, along with significantly less production of protective AMPs. This suggests that IL-17 and AMPs play an important role in VVC immunity. Based on the limited studies conducted, it appears that instead of being neutrophil-dependent, protection against VVC is reliant on extrinsic factors such as AMPS and the maintenance of an intact epithelial barrier and a balanced vaginal microbiota (44). Overall, studies regarding the role of IL-17 during VVC are inconclusive, and further research is required to determine if the function of IL-17 is protective, as observed at other sites of candida infection.

## Bacterial Infections

### Gonorrhoeae

Gonorrhea is an acute purulent genital tract infection caused by the Gram-negative bacterium, *Neisseria gonorrhoeae* (*N. gonorrhoeae*) (45). It is estimated that there are 78 million new cases of gonorrhea which occur globally each year (46), and due to the lack of a viable vaccine and the emergence of multi-drug resistant strains, *N. gonorrhoeae* is considered a serious infectious threat (46, 47). Rates of infection are higher in women compared to men, and gonorrhea infection can lead to further negative health outcomes in women including pelvic inflammatory disease (PID), infertility and ectopic pregnancy (48). Furthermore, if left untreated, infection with *N. gonorrhoeae* can enhance the transmission and acquisition of HIV by up to 5-fold (49).

*N. gonorrhoeae* is primarily an extracellular bacterium that induces a pro-inflammatory response consisting of the cytokines IL-6, IL-1β and tumor necrosis factor-alpha (TNF-α), as well as an influx of neutrophils (47). Interestingly, these cytokines are also linked to Th17 differentiation. As such, IL-17 levels have been reported to be elevated in individuals infected with *N. gonorrhoeae* (50, 51). The mouse model of gonorrhea, which requires mice to be treated with E2 prior to infection (52, 53), also demonstrates a strong Th17 response following infection. In this model, infection persists in rodents for about 10-20 days, after which the bacteria are cleared. Over the past several years, M.W. Russell and colleagues have extensively used the mouse model to better understand the role of IL-17 during gonorrheal infection. In initial studies, Feinen et al. (45) showed that IL-17 was critical for controlling early gonorrheal infection *in vivo*, as blocking IL-17 or preventing IL-17RA signalling in mice resulted in prolonged infection, along with significantly diminished neutrophil recruitment. Further, Russell and Feinen (54) reported that unlike IL-17, IL-22 does not appear to impact *N. gonorrhoeae* infection *in vivo*, suggesting IL-17 specifically, but not other Th17-related cytokines, is critical for bacterial clearance.

Interestingly, *N. gonorrhoeae* infection does not elicit a strong Th1 or Th2 response in mice (45) and although *N. gonorrhoeae* induces local inflammation, there is no acquired immunity or immunological memory established (53, 55). These findings show that primary infection does not lead to substantial or sustained antibody responses, and mice can be re-infected with the same strain of bacteria without displaying enhanced resistance such as elevated antibodies or enhanced CD4+ T cell responses (53). These observations closely reflect known features of the human immune response to uncomplicated *N. gonorrhoeae* infection, where there is limited humoral and T cell immunity, even with recurrent infection (56, 57). This led to the theory that *N. gonorrhoeae* selectively elicits Th17-dependent innate responses that it can overcome, including neutrophil recruitment and upregulation of AMP production, while suppressing Th1/Th2-driven adaptive immunity that may be able to protect against subsequent infection (45, 58–60). This would imply that Th17 immunity is manipulated by *N. gonorrhoeae* and used to evade host mechanisms of protection. In this regard, further work by Liu et al. has shown that the absence of protective Th1 responses can be attributed to the production of TGF-β, which occurs following *N. gonorrhoeae* infection and skews immune responses towards Th17-mediated immunity (59, 60). Together, these studies suggest that *N. gonorrhoeae* actually suppresses adaptive immunity by upregulating the production of immunosuppressive cytokines, TGF-β and IL-10. Liu et al. demonstrated that by blocking TGF-β, it is possible to reverse this host immune response and enable the development of protective anti-gonococcal immunity consisting of Th1-driven responses, the presence of anti-gonococcal IgG and IgA antibodies, establishment of immunological memory and enhanced clearance of bacteria (59, 60). Additional work is needed to understand how to better direct immunity during infection with *N. gonorrhoeae* to maximize protection, as well as to better understand how to leverage IL-17 immunity in this effort.

### Chlamydia

*Chlamydia trachomatis* (*C. trachomatis*) is an intracellular human bacterium that causes the most common bacterial sexually transmitted disease worldwide, with over 250 000 new infections contracted daily (61). Like many other STIs, women have disproportionately higher rates of chlamydia infection prevalence globally (62). Although antibiotics can be used to treat chlamydial infection, more than 70% of women show no signs or symptoms of active infection (63). As a result, untreated infections can cause significant reproductive tract pathology in women leading to the development of conditions such as PID, chronic pelvic pain and infertility. *C. trachomatis* is also the cause of preventable blindness (trachoma) in developing countries (64, 65).

Although IL-17 has been shown to be protective against extracellular pathogens, the role of IL-17 in protection against intracellular bacterial pathogens, including chlamydia, is less clear. Multiple mouse models for chlamydia have been developed over the past two decades and studies have provided extensive information regarding chlamydia pathogenesis, immune response and vaccine design. As with most animal models of infectious disease, there are differences between human and murine chlamydia infections which should be considered when extrapolating findings (66). For instance, similar to other models involving genital pathogens, the hormonal microenvironment of the murine FRT has to be manipulated in order for infection to occur. In the chlamydia models, mice require pre-treatment with progesterone to enhance susceptibility to infection. It is well known that changing the hormonal microenvironment alters the structural physiology of the FRT epithelium and may also influence the function of the immune cells present (67). Another difference is that in mice, disease develops after a single exposure to bacteria, after which infection is often cleared. This contrasts what occurs in humans, where secondary infections are often required to drive significant pathology and long-term chronic infections are common. Furthermore, different species of chlamydia display tropism for specific hosts (68). While human urogenital *C. trachomatis* strains can be used to infect mice, the most common murine model of genital chlamydia infection uses the mouse-adapted *C. muridarum* pathogen. Intravaginal infection with *C. muridarum* evades murine cell-autonomous immune mechanisms and establishes a self-resolving genital infection, whereas infection with human *C. trachomatis* is rapidly cleared from the murine FRT and fails to establish productive infection or induce pathogenic immune responses. As such, the *C. muridarum* genital infection model is more amenable to the study of immune mechanisms and appears to replicate many aspects of human infection. Studies have shown that *C. muridarum* first infects the vaginal and cervical epithelial cells, after which it ascends the reproductive tract and causes upper reproductive tract pathology, similar to chlamydia-associated disease sequelae observed in women infected with *C. trachomatis* (69, 70). The infection in mice is resolved after approximately 4-6 weeks and results in long-lived adaptive immunity that protects against re-infection (71). It has been shown that Th1 cells and interferon gamma (IFN-γ) are critical for protection against primary genital *C. muridarum* infection (72), while CD8+ T cell responses and antibody responses are important for protection against re-infection (73–78).

The role of IL-17 during chlamydial infection has been extensively studied in the lungs, where it has been shown that in the absence of IL-17, there is greater replication of bacteria and decreased survival of infected mice (79). Further mechanistic studies have demonstrated that IL-17 appears to be necessary for DC priming of Th1 immunity in the lungs, without which there is compromised bacterial clearance (80). However, in the context of genital tract infection with *C. muridarum*, the role of IL-17 is less clear. For instance, some studies have shown that the importance of IL-17 in mediating protection against chlamydia is negligible. A study by Scurlock et al. (81) showed that although IL-17RA deficient (IL-17RA-/-) mice demonstrated reduced IFN-γ production in the lymph nodes and decreased neutrophil influx into the FRT, mice were still able to resolve primary *C. muridarum* infection normally and showed no differences in pathology compared to WT mice. Instead, both macrophage influx and TNF-α production was increased in the absence of IL-17, suggesting a compensatory mechanism to control infection. Likewise, Frazer et al. (82) showed that in the absence of IL-23, where chlamydia-specific Th17 responses were absent, mice exhibited normal susceptibility to genital infection and regular development of oviduct pathology.

In contrast, other findings suggest that IL-17 plays a pathogenic role during genital chlamydial infection. In a study by Andrew et al. (64), they found that the duration and magnitude of *C. muridarum* infection was significantly lower in IL-17 deficient (IL-17A-/-) mice compared to WT mice. In the absence of IL-17, they also noted decreased inflammatory pathology, which was related to significantly reduced recruitment of neutrophils and macrophages into the oviduct tissues. Interestingly, others have also shown that oviduct pathology observed following infection is associated with the infiltration of neutrophils into the FRT, as well as the production of inflammatory mediators and factors involved in tissue-remodeling such as matrix metalloproteases; all of which are mediated by IL-17 (83–86). Human studies have similarly associated greater neutrophil activation with increased disease progression in women infected with genital *C. trachomatis* (87) and suggested a role for IL-17 during infection, as cervical washes from infected women had 5-fold higher levels of IL-17 compared to uninfected controls (88). Altogether, studies regarding the importance of IL-17 during chlamydial infection are conflicting. This may be due to the fact that the strains of mice used in different studies vary, and it is well known that the mouse strain used for chlamydia infection may impact outcomes, including duration of infection, degree of upper genital tract infection, severity of infection-induced pathology and immune responses generated (71). As such, further work is required to better elucidate the role of IL-17 in the context of genital chlamydia infection.

## Viral Infections

### HIV

HIV (human immunodeficiency virus) infection in the FRT leads to the rapid depletion of local CD4+ T cells, as well as viral dissemination throughout the body (89). It is a deadly virus if left untreated and can lead to the development of acquired immunodeficiency syndrome (AIDS). There are currently close to 38 million individuals living with HIV, with approximately 1.7 million new infections occurring each year (90). Women account for more than 50% of infections, and young women between the ages of 15–24 are particularly susceptible to infection (90). Although the risk of vaginal transmission is considered low, estimates indicate that 40% of HIV infections are initiated in the FRT (91). Additionally, infection with HIV is often associated with increased susceptibility to other sexually transmitted infections (STIs).

The role of IL-17 during HIV infection has not been comprehensively described, however, Th17 cells are important for HIV pathogenesis. HIV preferentially replicates in activated T cells, specifically CD4+ T cells (92), and the expression of the mucosal integrin α4β7 and HIV co-receptor CCR5 by activated T cells also increases susceptibility (93, 94). As such, Th17 cells, which are activated and terminally differentiated cells that express high levels of α4β7 and CCR5, are considered preferential target cells for HIV infection (95–100). *In vitro* and *ex vivo* studies using human cells and *in vivo* studies using a macaque model with the simian variant of the virus (SIV), have all shown that Th17 cells are target cells for HIV infection in mucosal tissues. For instance, Rodriguez-Garcia et al. (101) examined the phenotype and susceptibility of primary CD4+ T cells isolated from endometrium, endocervix, and ectocervix, to HIV infection *ex vivo*. They found that Th17 cells were the primary CD4+ T cell population which expressed HIV receptors CCR5 and CD90, and that these cells were the most susceptible to HIV infection *in vitro*. Likewise, several studies using samples collected from HIV-infected Kenyan women have also shown that Th17 cells are preferentially targeted by HIV, as Th17 cells were significantly depleted in infected individuals (93, 100, 102). Additionally, Boily-Larouche et al. (102) described a highly activated subset of CD4+ T cells in the FRT of HIV-infected female sex workers (FSWs) from Kenya, which expressed CD161 and differentiated into Th17 cells. These cells were found to express multiple HIV susceptibility markers and were severely depleted in HIV-infected FSWs, compared to uninfected FSWs. Similarly, McKinnon et al. (99) collected cervical cytobrush specimens from FSWs in Kenya and found that cervical IL-17+ CD4+ T cells preferentially co-expressed HIV receptors α4β7 and CCR5. Furthermore, these cervical Th17 cells were significantly depleted following HIV infection, suggesting they may serve as key target cells during HIV infection. Finally, in the macaque model of SIV infection, Stieh et al. (103) found that Th17 cells (CCR6+ CD4+) were also preferentially infected by SIV, and they showed that most SIV-infected cells expressed the master Th17 transcriptional regulator, ROR-γt.

Interestingly, Th17 and Th22 cells that make IL-17 and IL-122 play an important role in the renewal and maintenance of the mucosal epithelial barrier, which is known to be critical in protecting against HIV infection in both the gut and FRT (98, 104, 105). Several components of the mucosal epithelial barrier help mediate protection against HIV infection, including the physical composition of the barrier, the presence of immune cells, cytokines and antimicrobial factors, and the interactions between the barrier and the local microenvironment, including mucus and host microbiota (106). As such, the loss of Th17 cells may have a direct impact on the integrity of mucosal barrier and consequently impact further viral transmission and/or pathogenesis (107). This has been shown in the gut, where the depletion of Th17 and IL-22 producing T cells during chronic HIV infection has been associated with damage to the epithelial barrier and subsequent microbial translocation (108, 109). It is possible Th17 cells may be playing a similar protective role in the FRT during HIV infection, however more work is needed to elucidate this potential mechanism.

### HSV-2

Herpes simplex virus type 2 (HSV-2), the primary virus causing genital herpes, is one of the most common STIs worldwide, with over 400 million individuals infected globally and approximately 17 million new infections occurring each year (110). Rates of infection are especially alarming in sub-Saharan Africa, where prevalence is as high as 80% amongst women between the ages of 15-49 (110). HSV-2 first infects genital epithelial cells, and then travels *via* retrograde transport along nerve axons to the dorsal root ganglia, where it establishes life-long latency (111). The neuronal cells act as a reservoir for the latent virus, which can be reactivated due to factors including stress and hormonal changes. Reactivation results in anterograde transport of the virus, resulting in productive replication in the FRT (112). Along with the development of painful genital ulcers, HSV-2 infection is associated with a 2- to 3-fold increased risk of HIV-1 acquisition and up to a 5-fold increase in transmission of HIV-1 (113, 114).

Unlike the role of IL-17 during bacterial and fungal infections at mucosal sites, very limited studies have examined the importance of IL-17 in viral infections, especially in the context of the FRT and HSV-2. As such, this has been an area of interest in our lab and we have focused extensively on the role of both innate and adaptive production of IL-17 in the FRT, along with the role it plays during viral infection with HSV-2. Additionally, we have examined how factors in the FRT microenvironment, such as female sex hormones and the vaginal microbiota, effect IL-17 production in the genital mucosa.

Our work over the past few years has provided significant insight regarding how IL-17 modulates critical anti-viral T cell responses in the FRT. While it is well established that anti-viral protection against HSV-2 in the mouse model of infection is largely mediated by Th1 responses (115–117), our findings are the first to show that Th17 responses are also important in the anti-viral immune response to HSV-2 infection (118–120). We directly examined the role of IL-17 in anti-viral protection against HSV-2 and found that compared to WT controls, IL-17A-/- mice immunized intravaginally or intranasally were more susceptible to HSV-2 challenge (119). IL-17A-/- mice had decreased survival, greater viral shedding, and more severe genital pathology post-challenge. Interestingly, we found that IL-17 played an important role in enhancing anti-viral Th1 responses in the FRT, as IL-17A-/- mice had impaired Th1 cells responses post-challenge (119) and mechanistically, this was associated with impaired Th1 priming by vaginal DCs (118). Taken together, these studies have demonstrated that IL-17 is critical for inducing an efficient Th1 immune response following HSV-2 immunization, resulting in effective protection against HSV-2.

While the presence of innate IL-17 has been implicated in the amplification of Th17 responses in other mucosal tissues (121–123), the significance of IL-17 produced by innate or innate-like sources in the FRT and its influence on Th17 immunity in the vaginal mucosa is less understood. Thus, we also investigated the how innate IL-17 in the FRT might induce adaptive Th17 responses (124). Our findings support results seen in other mucosa and demonstrate that innate IL-17 produced in the FRT is also important for inducing Th17 responses. Furthermore, our observation that vaginal DCs from IL-17A-/- mice produced lower amounts of IL-1β compared to WT DCs, suggested a mechanism in which innate IL-17 induces vaginal DCs to prime Th17 responses *via* IL-1β (124). Additionally, consistent with previous findings by Kim et al. (125), we showed that γδ+ cells are the primary source of innate IL-17 in the FRT under homeostatic conditions (124). We extended these findings to show that multiple factors found within the vaginal microenvironment, influence innate IL-17 production by γδ+ cells. We found that E2 treatment resulted in significantly greater proportions of IL-17-producing γδ+ T cells and that germ-free mice had significantly lower proportions (124). These results provide insight on how innate IL-17 can influence immune responses against infections in the FRT, as well as ways in which its production can be modulated.

As mentioned previously, the hormonal microenvironment can influence immune responses and disease outcomes in the FRT. For the past decade we have investigated the influence of E2 on immune responses in the FRT and found that E2 increases protection against HSV-2 infection, although the underlying immunological mechanisms remained unclear. Recently, we showed that better protection in E2-treated mice coincided with earlier recruitment and higher proportions of Th1 and Th17 cells in the FRT following either HSV-2 immunization (intravaginally or intranasally) (120) and/or challenge (118). This included greater establishment of tissue-resident memory CD4+ T cells in the FRT, which play a critical role against re-exposure to pathogens (120). Together with the reduced protection shown against HSV-2 challenge in E2-treated IL-17A-/- mice, these findings suggested that E2-mediated protection against HSV-2 is driven by the induction of Th17 responses, likely mediated by increased IL-1β production by vaginal CD11c+ DCs in the presence of E2. Thus, a novel and critical anti-viral role for IL-17 in the FRT has emerged through our work.

## Conclusion

**Figure 2:**
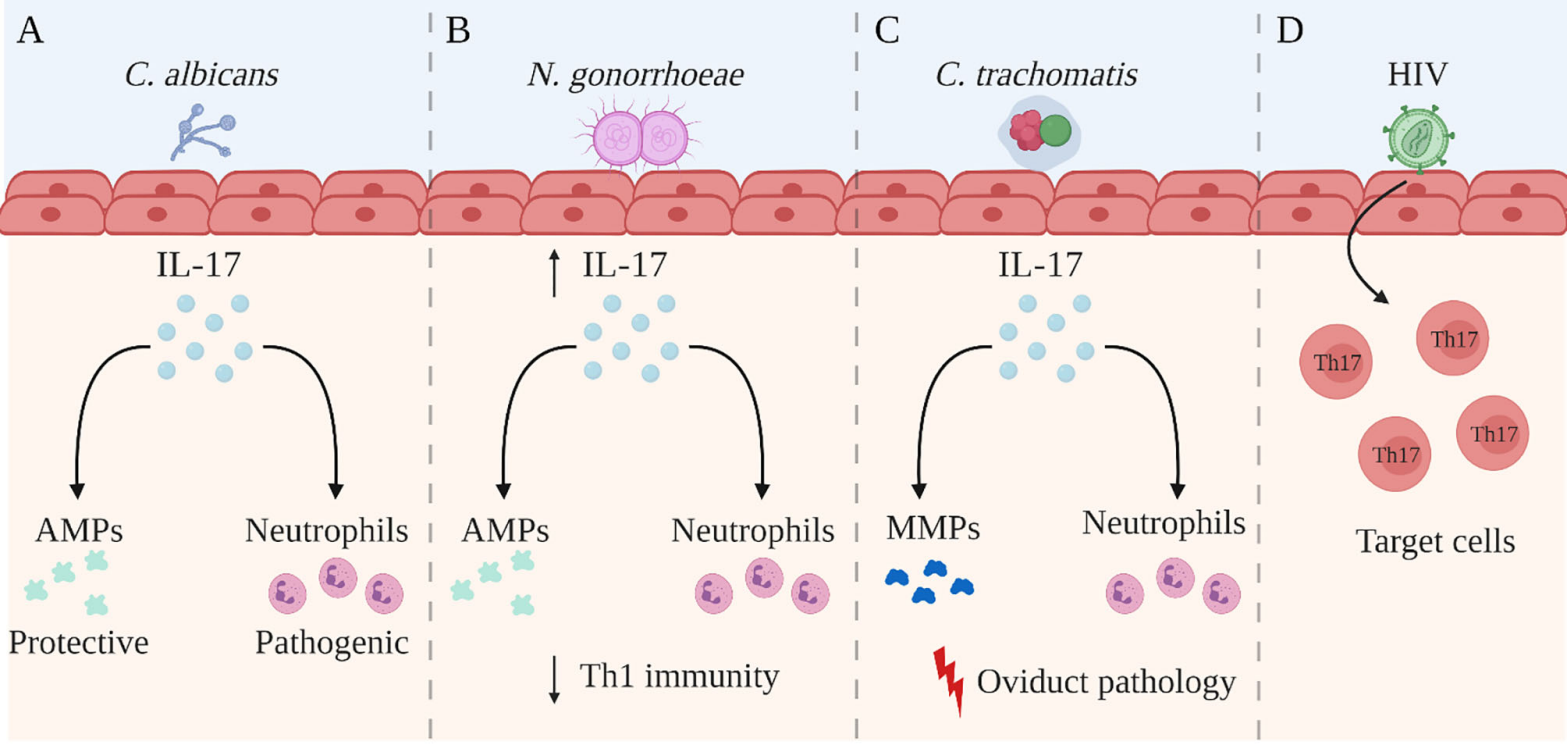
Summary of the role of IL-17 in the female reproductive tract. The role of IL-17A (IL-17) in the female reproductive tract (FRT) is disease-specific and is not yet completely understood. Studies have reported the following regarding the role of IL-17 during genital infection with Candida albicans (*C. albicans*), *Neisseria gonorrhoea* (*N. gonorrhoeae*), *Chlamydia trachomatis* (*C. trachomatis*), and human immunodeficiency virus **(A)** IL-17 is involved in recruiting neutrophils and inducing the production of anti-microbial peptides (AMPs) during *C albicans* infection. While AMPs have shown to be protective against *C albicans* infection in the FRT, neutrophils may be causing more damage than protection. **(B)** *N. gonorrhoeae* infection in the FRT selectively induces a robust Th17 response but reduced protective Th1 immunity, allowing evasion of host mechanisms of protection. However, the elevated IL-17 production has been shown to be important for controlling early gonococcal infection, as well as for recruiting AMPs and neutrophils. **(C)** During *C trachomatis* infection in the FRT, the recruitment of neutrophils and the production of matrix metalloproteases (MMPs) by IL-17 has been associated with greater oviduct pathology. **(D)** HIV preferentially infects activated Th17 cells which express CD4 and high levels of HIV co-receptors. Created with BioRender.

**Figure 3:**
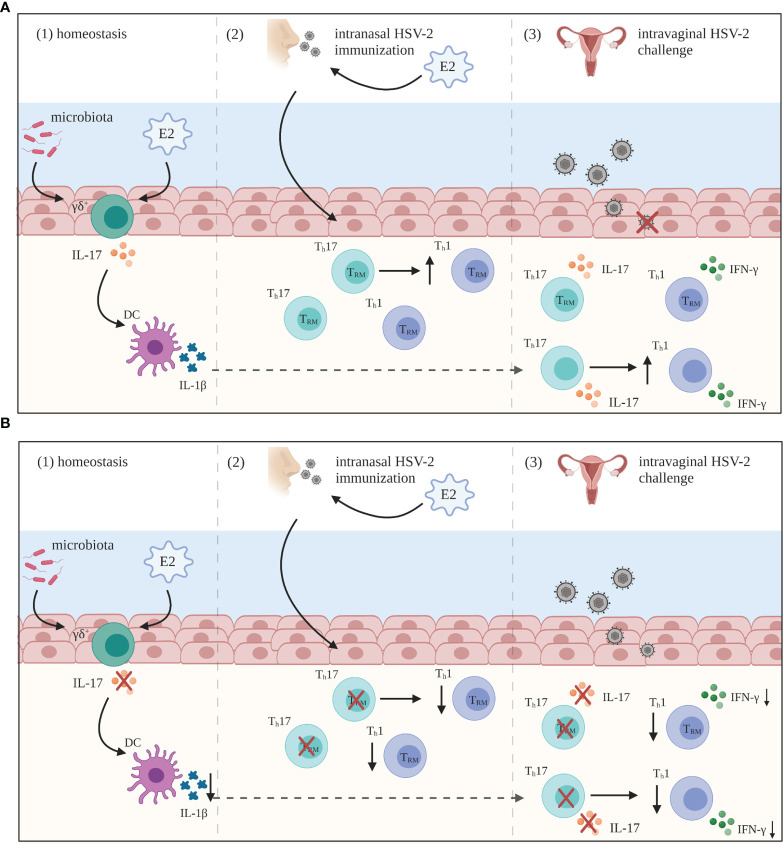
Summary of IL-17 mediated anti-HSV-2 immunity in the FRT. **(A)** (1) During periods of homeostasis, IL-17 is constitutively produced by innate lymphocyte populations, primarily gamma delta (γδ+) T cells, and regulated by estradiol (E2) and commensal microbiota. This innate IL-17 is important for the induction of Th17 responses primed by vaginal dendritic cells (DCs). (2) Following intranasal HSV-2 immunization under the influence of E2, IL-17 is important for the establishment of Th1 tissue-resident memory (TRM) cells in the female reproductive tract (FRT). (3) These Th17 and Th1 TRM cells are then able to protect against subsequent HSV-2 challenge. Additionally, IL-17 is critical for inducing IFN-γ+ CD4+ T cell recall responses in the FRT post-challenge. **(B)** In the absence of IL-17, there is reduced Th17 immunity generated (1), and overall anti-viral Th1 responses are significantly lowered both post-immunization (2) and post-challenge (3), even in the presence of E2. As a result, there is less protection generated against HSV-2 infection in the FRT. Created with BioRender.

The understanding of IL-17 and its role in immune processes has advanced considerably over recent years. While IL-17 has been shown to play a key role in the maintenance of tissue integrity and the generation of protective immunity against extracellular pathogens such as bacteria and fungi, the pro-inflammatory nature of IL-17 has also been associated with excessive inflammation and immunopathology. Within the context of the FRT, findings from several research groups have demonstrated the importance of IL-17 function which goes beyond merely acting as an inflammatory cytokine. The relevance of IL-17 has been demonstrated in bacterial, fungal and viral infections within the FRT, where innate and adaptive production of IL-17 is involved in a variety of immunomodulatory processes including neutrophil recruitment, DC regulation and Th1 modulation (**Figure 2**). However, it has become increasingly clear that the overall function of IL-17 is highly contextual, depending on both the pathogen involved and the site of infection. For instance, studies examining *C. albicans* infection in the FRT have demonstrated that unlike the clear protective effects of IL-17 observed in chronic mucocutaneous and oropharyngeal candidiasis, the function of IL-17 during vulvovaginal candidiasis remains inconclusive. Similarly, while IL-17 has shown to be protective against chlamydial infection in the lungs, the role of IL-17 is less clear in the FRT. Some findings suggest IL-17 does not impact disease outcomes following genital chlamydial infection, while other studies have demonstrated a pathogenic role. This highlights the tissue-specific function of IL-17, as well as the necessity to further study the extent to which IL-17 is required in facilitating immunity during these infections. Additionally, some genital tract pathogens such as HIV and *N. gonorrhoeae* appear to leverage IL-17-mediated responses to their advantage by either targeting Th17 cells to promote infection or by using these responses to suppress the generation of protective adaptive immunity, respectively. Yet, our work has shown that IL-17 seems to have a broader protective role in the context of HSV-2 infection in the FRT. We have recently demonstrated that IL-17 modulates vaginal DCs to induce greater Th1 and Th17 immunity in the FRT and is also involved in increased establishment of memory T cells post-immunization, which results in greater protection against genital HSV-2 infection (**Figure 3**). Our studies have also emphasized the critical role of hormones in regulating levels of IL-17 within the reproductive mucosa. Altogether, these studies point to the need to better understand the dual role and balance between IL-17-mediated protection and pathology before considering therapeutically modulating the IL-17 pathway. Further research is needed to gain a better understanding of the underlying factors including the tissue microenvironment that regulate IL-17 and Th17 immune responses. Consequently, incentives directed towards developing vaccines or therapies for these pathogens must consider whether supporting or inhibiting IL-17 responses in the FRT will help shape the immune response towards a more robust protective phenotype that can help resist these pathogens.

## Author Contributions

The concept for this review was determined by CK. The first draft was written by PB. All authors participated in the editing and revision of the manuscript. PB provided the initial production of the figures and their revision. All authors participated in the final revision of the manuscript and approved the final version.

## Funding

This research was funded by operating grants from the Canadian Institutes of Health Research (CIHR) to CK (FRN #93615) and an Applied HIV Research Chair Award to CK from the Ontario HIV Treatment Network (OHTN) (AHRC #779).

## Conflict of Interest

The authors declare that the research was conducted in the absence of any commercial or financial relationships that could be construed as a potential conflict of interest.

## Publisher’s Note

All claims expressed in this article are solely those of the authors and do not necessarily represent those of their affiliated organizations, or those of the publisher, the editors and the reviewers. Any product that may be evaluated in this article, or claim that may be made by its manufacturer, is not guaranteed or endorsed by the publisher.
